# Editorial: Emerging opportunities in congenital cardiac surgery

**DOI:** 10.3389/fcvm.2023.1343354

**Published:** 2023-12-07

**Authors:** Paul Philipp Heinisch, Sebastian Michel, Daniel Zimpfer, Jürgen Hörer

**Affiliations:** ^1^ Department of Congenital and Pediatric Heart Surgery, German Heart Center Munich, Technische Universität München, Munich, Germany; ^2^ Division of Congenital and Pediatric Heart Surgery, University Hospital of Munich, Ludwig-Maximilians-Universität, Munich, Germany; ^3^ Department of Cardiac Surgery, Medical University of Vienna, Vienna, Austria

**Keywords:** congenital cardiac, heart surgery, innovation, opportunity, technology

**Editorial on the Research Topic** Emerging Opportunities in Congenital Cardiac Surgery

Congenital Heart Disease (CHD) continues to be the most prevalent congenital anomaly on a global scale, impacting approximately 1% of neonates. In recent decades, significant progress has been made in the diagnosis and treatment of congenital heart disease (CHD), resulting in significant changes in the field of congenital cardiac surgery. This review examines the aforementioned advancements, assessing their influence on patient outcomes and the dynamic characteristics of CHD treatment.

## Diagnostic progress: a leap forward in early detection

The development in diagnostic tools, especially in echocardiography, has been a cornerstone in CHD management. The early and accurate diagnosis of conditions such as hypoplastic left heart syndrome (HLHS) is now possible, thanks to the ability to detect restrictive or intact atrial septum. This early detection is crucial as it enables prompt surgical intervention, significantly improving patient outcomes.

## Surgical interventions: palliative yet life-enhancing

Unlike many other medical conditions, the surgical intervention for CHD is often palliative rather than reparative. However, this does not diminish its value. The advancements in pre and post-operative care, alongside minimally invasive surgical techniques, have dramatically improved survival rates. It's a notable achievement that over 85% of CHD neonates are now expected to reach adulthood. This improvement, however, brings new challenges, including an increased number of adults requiring additional operations, often associated with complications such as endocarditis.

## Interventional cardiology: a new era in CHD management

The field is witnessing a paradigm shift with the growing preference for congenital heart interventions over conventional surgical approaches. Innovations like ductal stents are showing favorable outcomes compared to systemic-to-pulmonary artery shunting. This evolution underscores the need for closer collaboration between cardiac surgeons and interventional cardiologists to ensure optimal patient care.

## Emerging opportunities and research directions

Several emerging research areas hold promise for further improving CHD management:

1. Pleural and Mediastinal Effusions after the Extracardiac Total Cavopulmonary Connection: Heinisch et al. in “Pleural and mediastinal effusions after the extracardiac total cavopulmonary connection: Risk factors and impact on outcome” discuss the correlation between these effusions and various clinical factors, highlighting the risks and outcomes associated with the procedure.
2. Operability of Atrial Septal Defect with Borderline Pulmonary Vascular Resistance Index: Lilyasari et al. in their study “Operability of atrial septal defect with borderline pulmonary vascular resistance index: A study in developing country” delve into the perioperative outcomes and challenges in managing atrial septal defect under specific pulmonary conditions, offering insights into operative strategies in a developing country context.
3. Case Report: Heart Mate III for Systemic Right Ventricular Support in a Patient with Hypoplastic Left Heart Syndrome: Hanuna et al. present a case report detailing the use of Heart Mate III for systemic right ventricular support in a patient with hypoplastic left heart syndrome, exploring new frontiers in cardiac support technology.
4. Biogenic Polymer-Based Patches for Congenital Cardiac Surgery: In the study by Richert et al., “Biogenic polymer-based patches for congenital cardiac surgery: a feasibility study”, the authors examine the potential and practicality of using biogenic polymer-based patches in congenital cardiac surgery, contributing to the evolution of surgical materials.
5. Past, Present, and Future Options for Right Ventricular Outflow Tract Reconstruction: Carrel in “Past, present, and future options for right ventricular outflow tract reconstruction” reviews the historical, current, and potential future techniques and materials used for reconstructing the right ventricular outflow tract, a critical aspect of congenital heart surgery.
6. Contemporary Sequential Segmental Approach to Congenital Heart Disease Using Four-Dimensional Magnetic Resonance Imaging with Ferumoxytol: Yoo et al. discuss a novel imaging approach in “Contemporary sequential segmental approach to congenital heart disease using four-dimensional magnetic resonance imaging with ferumoxytol: an illustrated editorial”, providing a new perspective on diagnostic imaging in CHD.
7. Successful Surgical Repair in an Older Adult with Supracardiac Total Anomalous Pulmonary Venous Connection (Wang et al.): The case report demonstrates successful surgical repair in an older adult with supracardiac total anomalous pulmonary venous connection, a rare and complex procedure in this patient demographic, showing progress in surgical techniques and patient management.
8. Bilateral Lung Transplantation for Pediatric Pulmonary Arterial Hypertension (Jack et al.): This article covers the perioperative management and one-year follow-up of pediatric patients undergoing bilateral lung transplantation for pulmonary arterial hypertension, providing valuable insights into this challenging and rare procedure.
9. Beneficial Long-term Effect of the Atrial-Flow-Regulator Device in a Pediatric Patient with Idiopathic Pulmonary Arterial Hypertension and Recurring Syncope (Pattathu et al.): This case report discusses the long-term benefits of using the atrial-flow-regulator device in a pediatric patient with idiopathic pulmonary arterial hypertension and recurring syncope, offering a glimpse into innovative treatment options for managing complex cardiac conditions (9).
10. Central Venous Catheter Thrombosis Complicated by Chronic Thromboembolic Disease/Pulmonary Hypertension in Two Children Requiring Parenteral Nutrition (Hanuna et al.): The study details the complications of central venous catheter thrombosis leading to chronic thromboembolic disease and pulmonary hypertension in children requiring parenteral nutrition, highlighting the challenges in managing such intricate clinical scenarios (10)

The aforementioned studies collectively emphasise the fluid and progressive nature of managing and treating congenital heart disease (CHD), thereby highlighting the continuous research and collaborative endeavours in this domain with the objective of enhancing patient outcomes.

The objective of this research topic is to present a comprehensive survey of contemporary and developing surgical methodologies for patients with congenital heart disease (CHD) across all age groups. The importance placed on interdisciplinary collaboration between cardiac surgeons and interventional cardiologists is a notable component, underscoring the necessity of an integrated approach for achieving the most favourable outcomes for patients.

## Conclusion

The field of congenital cardiac surgery and management of CHD has seen transformative changes over the past decades. These advancements have not only improved survival rates but also opened new avenues for research and treatment. The future of CHD management lies in continued innovation, interdisciplinary collaboration, and an unwavering commitment to understanding and addressing the complex needs of this high-risk patient population. The ongoing research and development in this field are pivotal in shaping a future where CHD patients can lead longer, healthier lives.

